# A Dietary Antioxidant Formulation Ameliorates DNA Damage Caused by γ-Irradiation in Normal Human Bronchial Epithelial Cells In Vitro

**DOI:** 10.3390/antiox11071407

**Published:** 2022-07-20

**Authors:** J. P. Jose Merlin, Sabateeshan Mathavarajah, Graham Dellaire, Kieran P. J. Murphy, H. P. Vasantha Rupasinghe

**Affiliations:** 1 Department of Plant, Food and Environmental Sciences, Faculty of Agriculture, Dalhousie University, Truro, NS B2N 5E3, Canada; josemerlinj@dal.ca; 2 Department of Pathology, Faculty of Medicine, Dalhousie University, Halifax, NS B3H 1X5, Canada; smathavarajah@dal.ca (S.M.); dellaire@dal.ca (G.D.); 3 Department of Medical Imaging, Faculty of Medicine, University of Toronto, Toronto, ON M5T 2S8, Canada; kieran.murphy@uhn.ca

**Keywords:** cancer, chemoprevention, γ-irradiation, carcinogen, DNA damage, flavonoids

## Abstract

Antioxidants can be used as radioprotectants to reduce DNA damage due to exposure to radiation that could result in malignancies, including lung cancer. Mortality rates are consistently higher in lung cancer, which is usually diagnosed at later stages of cancer development and progression. In this preliminary study, we examined the potential of an antioxidant formulation (AOX2) to reduce DNA damage using a cell model of human normal bronchial epithelial cells (BEAS-2B). Cells were exposed to γ-irradiation or smoke-related hydrocarbon 4[(acetoxymethyl)nitrosamino]-1 (3-pyridyl) 1-butanone (NNKOAc) to induce DNA damage. We monitored intracellular reactive oxygen species (ROS) levels and evidence of genotoxic damage including DNA fragmentation ELISA, γ-H2AX immunofluorescence, and comet assays. Pre-incubation of the cells with AOX2 before exposure to γ-irradiation and NNKOAc significantly reduced DNA damage. The dietary antioxidant preparation AOX2 significantly reduced the induction of the tumor suppressor protein p53 and DNA damage-associated γ-H2AX phosphorylation by radiation and the NNKOAc treatment. Thus, AOX2 has the potential to act as a chemoprotectant by lowering ROS levels and DNA damage caused by exposure to radiation or chemical carcinogens.

## 1. Introduction

Lung cancer is the most diagnosed and leading cause of cancer-related death for both men and women [1,2]. Some environmental and lifestyle factors, such as mutagenic chemicals, ionizing radiation (IR), endogenous reactive oxygen species (ROS), and unresolved intermediates of physiologic topoisomerase and nuclease reactions, are linked to the initiation of lung cancer [3]. In addition, cigarette smoking contributes to 85% to 90% of lung cancer cases [4]. Low-dose ionization irradiation can also lead to significant lung and skin damage through the activation of transforming growth factor-beta (TGF-β) [5]. Radiation and carcinogens can induce single-strand breaks (SSBs), double-strand breaks (DSBs), as well as DNA adducts and base lesions [6]. Of these forms of DNA damage, DNA DSBs are the most dangerous, triggering cancer-causing chromosomal breaks, translocations, and other chromosome abnormalities [3]. If sufficient in number, DNA DSBs can result in cell death or the production of inflammatory cytokines that injure tissues and contribute to organ pathologies [7,8]. Among lung cancer treatments, ionizing radiation is often the most effective in suppressing the growth of non-small cell lung cancer [9,10].

Cigarette smoke contains a pro-carcinogenic hydrocarbon 4-[(methyl)nitrosamino]-1-(3-pyridyl)-1-butanone (NNK), which is converted into reactive metabolites by cytochrome P-450 enzymes. These metabolites can covalently bind to DNA, creating DNA adducts that cause DSBs [11]. Since NNK requires α-hydroxylation for metabolic activation, a derivative of NNK, 4-[(acetoxymethyl) nitrosamino]-1-(3-pyridyl)-1-butanone (NNKOAc), is commonly used in cell culture to simulate in vivo xenobiotic metabolism [12,13]. The detection of DSB by ataxia telangiectasia mutated (ATM) is the initial step in the DNA damage response (DDR). Activation of ATM results in the phosphorylation of the histone variant H2AX on Ser-139 (referred to as γ-H2AX) within the chromatin adjacent to the DNA DSB, which promotes the DDR through the DNA damage sensor Mre11/Rad50/Nbs1 (MRN) complex, as well as the accumulation of p53-binding protein-1 (53BP1) [14]. As well, the DDR involves the activation of checkpoint kinases and the tumor suppressor transcription factor p53 to facilitate cell-cycle arrest, which allows time for DNA repair, after which the cell cycle returns to normal if the DNA damage is not too extensive [15].

Radiation and many other carcinogens also damage DNA by generating reactive oxygen species (ROS) that induce DNA–DNA and protein–DNA adducts, intra/inter-strand crosslinks, ssDNA breaks, and base damage [16]. Dietary antioxidants have the potential to lower the risk of oxidative damage-mediated cancer development [17,18,19,20]. Plant-food flavonoids alone or in combinations with antioxidative vitamins have been shown to protect normal human bronchial epithelial cells (BEAS-2B) from NNKOAc-induced carcinogenesis [13,21]. For example, curcumin (Cur) and vitamin E quench ROS produced by exposure to acute benzo[a]pyrene (BaP) in lung epithelial cells [22]. Administration of specific plant natural substances inhibits carcinogenesis in healthy and high-risk individuals; thus, certain cancers are preventable through dietary supplements of antioxidants [23]. Dietary supplementation of flavonoids helped genoprotection when male Swiss mice were exposed to X-rays [24]. Flavonoids regulate all phases of the intricate process of carcinogenesis, which is linked to inflammation. Thus, flavonoids and other phytochemicals have been proposed to be used in the overall cancer management framework of predictive, preventive, and personalized medicine [25]. We investigated whether an antioxidant formulation called AOX2 that contains vitamin C (ascorbic acid; AA), vitamin B9 (folate), vitamin B12 (cyanocobalamin), vitamin E (α-tocopherol), α-lipoic acid, coenzyme Q10 (CoQ10), astaxanthin, zeaxanthin, quercetin (Q), and sodium selenite could help develop chemoprotective interventions for ROS-induced DNA damage caused by diagnostic scans employing ionizing radiation and smoke-related carcinogens by examining if it could protect against DNA damage caused by low-dose γ-radiation and the chemical carcinogen NNKOAc in BEAS-2B cells.

## 2. Materials and Methods

### 2.1. Chemicals, Kits, Antibodies, and AOX2 Formulation

LHC-9 growth medium for BEAS-2B cells was purchased from Thermo Fisher Scientific (Chelmsford, MA, USA). A COMET SCGE assay kit for comet assay was purchased from ENZO (New York, NY, USA). For γ-H2AX immunofluorescence studies, primary antibody anti-H2AX (S139) (#05-636) was obtained from Millipore (Etobicoke, ON, Canada) and secondary antibody Alexa Flour 594 donkey anti-mouse (#A-21203) was purchased from Life Tech (Carlsbad, CA, USA). p-p53 (#9286), γ-H2AX (#9718), and β-actin (#12620) were purchased from Cell Signaling Technology (Boston, MA, USA). 4-[(Acetoxymethyl) nitrosamino]-1-(3-pyridyl)-1-butanone (NNKOAc) was purchased from Toronto Research Chemicals (Toronto, ON, Canada). Ascorbic acid, folate, cyanocobalamin, α-tocopherol, α-lipoic acid, CoQ10, astaxanthin, zeaxanthin, quercetin, sodium selenite, curcumin, 3-(4,5-dimethylthiazol-2-yl)-5-(3-carboxy methyl phenyl)-2-(4-sulfophenyl)-2H-tetrazolium (MTS), phenazine methosulfate (PMS), and dichlorofluorescin diacetate dye (DCFH-DA) were purchased from Sigma-Aldrich (Oakville, ON, Canada). A cellular DNA fragmentation enzyme-linked immunosorbent assay (ELISA) kit for DNA fragmentation analysis was received from Roche Diagnostics (Berlin, Germany). The dietary antioxidant formulation (AOX2) consists of ascorbic acid, folate, cyanocobalamin, α-tocopherol, α-lipoic acid, CoQ10, astaxanthin, zeaxanthin, quercetin, and sodium selenite (2.5 µM each). Except for α-tocopherol, CoQ10, and sodium selenite, all of the compounds were dissolved in DMSO to make a 100 mM stock solution. The stock solutions of cyanocobalamin, astaxanthin, and zeaxanthin were 10 mM. Warm DMSO was used to dissolve α-tocopherol and CoQ10, while sodium selenite was dissolved in LHC-9 medium (100 mM).

### 2.2. Cell Culture 

BEAS-2B cells were purchased from the American Tissue Type Culture Collection (ATCC; CRL-9609; Manassas, VA, USA) and grown in LHC-9 medium at 37 °C in a humidified incubator with 5% CO_2_. Fibronectin (0.01 mg/mL), bovine collagen type I (0.03 mg/mL), and bovine serum albumin (0.01 mg/mL) dissolved in LHC-9 media were pre-coated on culture flasks (polystyrene T75) overnight. The passages (<10) were utilized for all experimental settings after the cells were grown to around 70% confluence.

### 2.3. Irradiation by Gammacell Irradiator

Low dose γ-irradiation was performed at room temperature for a defined time (1 Gy/15 s, 2 Gy/30 s, 4 Gy/60 s, 6 Gy/90 s, 8 Gy/120 s, and 10 Gy/150 s) using a Gammacell^®^ 3000 Elan (Best Theratronics Ltd., Kanata, ON, Canada). After irradiation of cells, incubation was continued at 37 °C for a further 24 h before harvesting by trypsinization and re-seeding the cells for all treatment.

### 2.4. Cell Viability by MTS Assay

The viability of BEAS-2B cells was determined as the metabolic activity using MTS assay [21] under various treatment conditions. Preliminary experiments with various low doses of γ-radiation (1 to 10 Gy) were performed to identify the non-cytotoxic dose for the evaluation of antioxidants. MTS reagent was applied to each well and incubated in the dark for 3 h. A microplate reader (Infinite^®^ 200 PRO, TECAN, Mannedorf, Switzerland) was used to measure absorbance at 490 nm. For each experiment, cells with DMSO medium served as the vehicle control and MTS reagent-containing medium without cells served as the blank.

### 2.5. Clonogenic Cell Survival Assay

The clonogenic cell survival assay was carried out as previously described [26]. Briefly, BEAS-2B cells (1.0 × 10^5^ cells/mL) were treated with 1 to 10 Gy of low dose γ-radiation for 24 h before being trypsinized and counted. They were re-seeded in a 60 mm dish, cultivated for 21 days, and colonies were recognized as survivors and counted after staining with 0.5% crystal violet. The survival rate was estimated after normalizing the results against equivalent non-irradiated cells.

### 2.6. Measurement of Intracellular ROS

BEAS-2B cell cultures were added with DCFH-DA dye, which was taken up by cells and hydrolyzed to DCFH, which may be oxidized by ROS to yield the fluorescent product dichlorofluorescein (DCF) [27]. After 3 h of pre-exposure to tested antioxidants, the cells were subjected to γ-irradiation or NNKOAc for 3 h. The vehicle control consisted of cells that only had DMSO medium (the same treatment conditions were used for all experiments). After the treatments, the cells were mixed with a final concentration of 5 μM DCFH-DA and incubated in the dark for 40 min. A plate reader (Infinite^®^ 200 PRO, TECAN, Mannedorf, Switzerland) was used to measure the fluorescence at an excitation wavelength of 485 nm and an emission wavelength of 535 nm.

### 2.7. DNA Fragmentation Analysis

In BEAS-2B cells, DNA fragmentation was assessed using a cellular DNA fragmentation ELISA kit. Bromodeoxyuridine (BrdU, 10 μM) was used to label cells (1 × 10^5^ cells/mL), and 100 μL of BrdU-labeled cells were treated as described previously (Section 2.6). After centrifugation at 250× *g* for 10 min, the cells were lysed with lysis buffer, and apoptotic DNA fragments in supernatants were collected for each sample. The samples (100 μL) were then transferred to 96-well flat-bottom microplates pre-coated with anti-DNA and incubated at 25 °C for 90 min. Microwave irradiation (500 W for 5 min) was used to denature the DNA, followed by the addition of 100 μL of anti-BrdU-POD conjugate solution for an additional 90 min of incubation. This was followed by three washes with wash buffer (1×) and the addition of 100 μL of substrate 3,3′,5,5′-tetramethylbenzidine (TMB) solution for color development. After 5 min, the stop solution (25 μL) was added, and the plates were read at 450 nm using a microplate reader (Infinite^®^ 200 PRO, TECAN, Mannedorf, Switzerland).

### 2.8. γ-H2AX Immunofluorescence Assay

By measuring γ-H2AX foci in BEAS-2B cells, the immunofluorescence assay was utilized to evaluate DNA damage at the histone protein level [28]. In a 6-well plate, 1 × 10^5^ cells were seeded on a coated coverslip and incubated for 24 h. After the treatments, the cells were rinsed completely in phosphate buffered saline (PBS) and fixed with 3.7% formaldehyde before being incubated in the dark for 20 min. The cells were then permeabilized for 15 min at room temperature with 0.5% Triton X-100 in PBS, followed by blocking with 4% BSA for 20 min. The cells were treated for 1 h at room temperature with the primary antibody (1:250), rinsed three times with PBS, and then incubated for 45 min with the secondary antibody (1:500). The cells were rinsed three times in PBS before being gently put onto the slides, mounted using wet-mounting media containing DAPI and sealed with nail polish. A fluorescence microscope was used to capture the fluorescent images (EVOSTM FLoid Imaging System, Bothell, WA, USA).

### 2.9. Comet Assay

The comet assay, also known as single-cell gel electrophoresis, was used to assess DNA damage [29]. Briefly, treated cells were mixed 1:10 (*v*/*v*) with molten low melting agarose, and 75 μL of each sample was pipetted onto the slide and incubated for 20 min at 4 °C in the dark. After 45 min in cold lysis buffer at 4 °C, the slides were submerged in alkaline solution for another 30 min in the dark. After, the slides were washed in 1× TBE buffer for 5 min and subjected to electrophoresis (1 V/cm for 10 min). The slides were soaked in 70% ethanol for 5 min, air-dried, then dyed with CYGREEN^®^ dye (1:1000) before being studied under fluorescence microscopy (EVOSTM FLoid Imaging System; Bothell, WA, USA). Comet assay software (http://casplab.com/download, accessed on 15 May 2022) was used to score the comets, and a minimum of 30 cells were quantified by measuring the % DNA tail moment.

### 2.10. Western Blotting

After the treatments, BEAS-2B cells were harvested and lysed in 1 × sodium dodecyl sulfate (SDS) lysis buffer (1 mM Tris–HCl (pH 6.8), 2% *w*/*v* SDS, 10% glycerol) on ice under reduced conditions. The Bradford test was used to determine the total protein concentration in each sample. On a 12% SDS-PAGE gel, 20 μg of protein samples were loaded and electro-transferred to a polyvinylidene difluoride (PVDF) membrane (Thermo Fisher, Mississauga, ON, Canada). The membrane was then blocked with a 5% non-fat milk solution for 1 h at room temperature, then probed with specific primary antibodies (p-p53 and γ-H2AX at 1:1000) for overnight incubation, washed, and probed again with respective secondary antibodies (1:2000) for 1 h, and then developed using enhanced chemiluminescence (ECL) based on Clarity™ and Clarity Max™ (Bio-Rad, ChemiDoc^TM^ MP, Hercules, CA, USA). Each band’s protein expression was normalized to its respective actin protein level, and relative protein expression was quantified for each experiment in comparison to untreated control bands.

### 2.11. Statistical Analysis

All the experiments were performed in triplicate (n = 3) three independent times and analyzed by one-way analysis of variance (ANOVA) by using Tukey’s post hoc test and two-tailed Student’s *t*-test by using GraphPad Prism 5 software (GraphPad software Inc., San Diego, CA, USA). Data were presented as mean ± standard deviation (SD) and *p* ≤ 0.05 was significant between experimental groups.

## 3. Results

### 3.1. Assessing the Sensitivity of Normal Bronchial Epithelial Cells to γ-Irradiation

**Figure 1:**
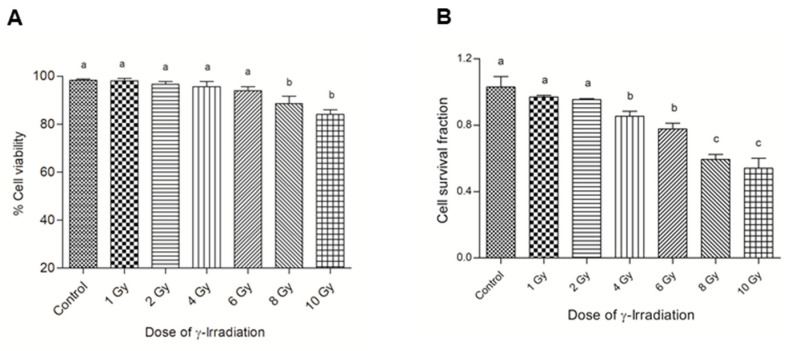
The viability (**A**) and survival fraction (**B**) of BEAS-2B cells after exposure to γ-irradiation. The cell viability was measured using the MTS assay. The survival fraction was determined using the clonogenic cell survival assay, where BEAS-2B cells were incubated for 24 h after irradiation. Then, the cells were re-seeded into 60 mm dishes and cultured for 21 days. Colonies were stained with crystal violet. Experimental values presented are mean ± SD of three independent experiments in triplicate. One-way analysis of variance was performed with Tukey’s pairwise comparison. Means that share the same letter are not significantly different at *p* ≤ 0.05.

To determine the optimal γ-irradiation dose for the cell model of DNA damage, first the MTS assay was employed to investigate the dose-dependent effects on cell viability (Figure 1A). The doses ranging from 1 to 6 Gy of γ-irradiation did not affect the cell viability as measured by MTS, while the doses of 8 and 10 Gy were significantly different when compared to other doses of γ-irradiation. However, clonogenic survival, which integrates both the ability of cells to divide post-irradiation and their survival, was significantly lower in BEAS-2B cells treated with high doses of γ-irradiation from 6 to 10 Gy (Figure 1B). Based on these observations, we chose 4 Gy γ-irradiation for the subsequent experiments in line with previous studies [26]. 

### 3.2. AOX2 Is Not Cytotoxic at Lower Concentrations

**Figure 2:**
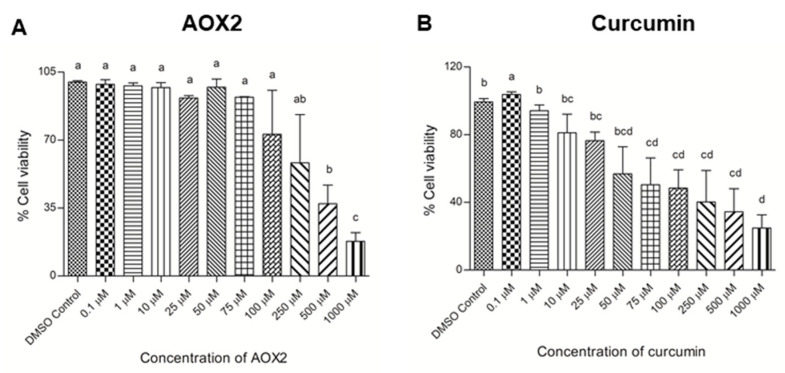
The viability of BEAS-2B cells against dose-dependent exposure to AOX2 (**A**) and curcumin (**B**) for 24 h. Experimental values are mean ± SD of three independent experiments in triplicate. One-way analysis of variance was performed with Tukey’s pairwise comparison. Means that share the same letter are not significantly different at *p* ≤ 0.05. Abbreviations: AOX2, dietary antioxidant formulation-2; DMSO, dimethyl sulfoxide.

AOX2 had no cytotoxic effect on BEAS-2B cells up to 100 μM, but at high concentrations of 250 μM and above, the cell viability reduced significantly (*p* ≤ 0.05) (Figure 2A). Curcumin caused over 20% loss of cell viability at and above 50 μM concentration (Figure 2B). In a prior work, we found that ascorbic acid had no cytotoxic impact, but higher concentrations of quercetin (1 mM) were cytotoxic to BEAS-2B cells (Merlin et al., 2021). Based on the above observations, 25 μM of AOX2 (2.5 μM of each component), ascorbic acid, quercetin, and curcumin were chosen for further studies. All of the treatments were compared to a DMSO control with ≤5% cytotoxicity level.

### 3.3. AOX2 Reduced the γ-Irradiation- and Carcinogen-Induced Intracellular ROS 

**Figure 3:**
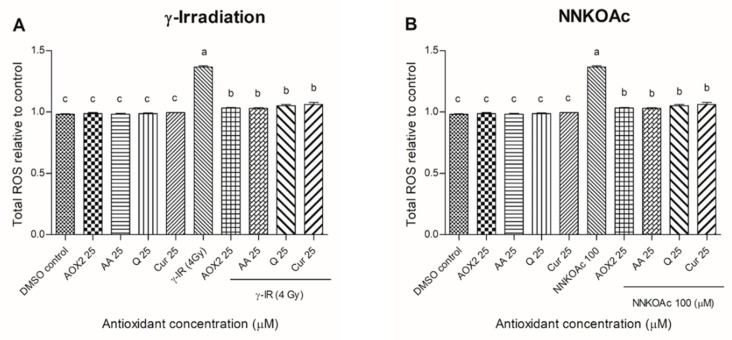
ROS production was reduced by pre-exposure to tested antioxidants in γ-irradiation (**A**) or carcinogen (**B**) induced BEAS-2B cells. Experimental values are mean ± SD of three independent experiments in triplicate. One-way analysis of variance was performed with Tukey’s pairwise comparison. Means that share the same letter are not significantly different at *p* ≤ 0.05. Abbreviations: AOX2, dietary antioxidant formulation-2; AA, ascorbic acid; Q, quercetin; Cur, curcumin; DMSO, dimethyl sulfoxide; γ-IR, γ-irradiation.

In a recent investigation, we found that 100 μM NNKOAc is the optimum concentration for use in this carcinogen-induced DNA damage cell model [13,21]. Employing the DCFH-DA dye ROS assay, we examined the impact of dietary antioxidants on lowering intracellular ROS levels in γ-irradiated- and carcinogen-exposed BEAS-2B cells (Figure 3). In comparison to the control, the γ-irradiated- and NNKOAc-treated BEAS-2B cells had 1.5-fold higher intracellular ROS. However, pre-exposure to AOX2 and tested reference antioxidants reduced ROS levels significantly (*p* ≤ 0.05) when compared to the control γ-irradiation- and NNKOAc-exposed cells.

### 3.4. AOX2 Ameliorates DNA Damage Caused by Carcinogenic Factors 

**Figure 4:**
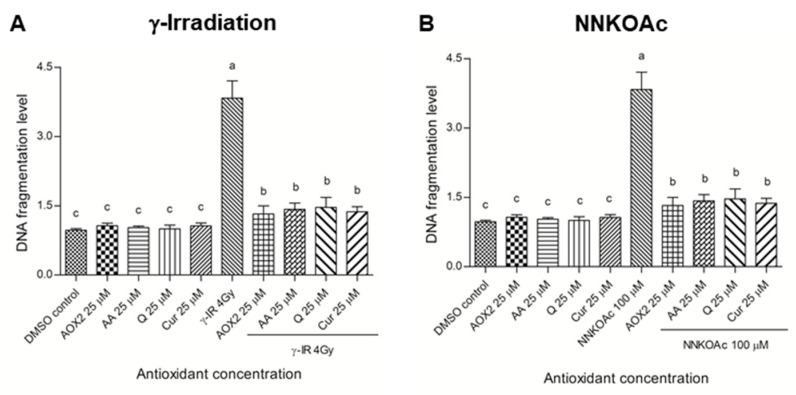
DNA fragmentation was reduced by the tested antioxidants in γ-irradiation (**A**) or carcinogen (**B**) induced BEAS-2B cells. DNA fragmentation was determined by ELISA. Experimental values are mean ± SD of three independent experiments in triplicate. One-way analysis of variance was performed with Tukey’s pairwise comparison. Means that share the same letter are not significantly different at *p* ≤ 0.05. Abbreviations: AOX2, dietary antioxidant formulation-2; AA, ascorbic acid; Q, quercetin; Cur, curcumin; DMSO, dimethyl sulfoxide; γ-IR, γ-irradiation.

An ELISA technique [30] was used to determine the amounts of DNA fragments in BEAS-2B cells (Figure 4). The γ-irradiation and NNKOAc exposure increased DNA fragmentation almost four-fold when compared to the DMSO control. AOX2 and tested antioxidants alone did not cause any DNA fragmentation. The pre-treatment with antioxidants significantly reduced (*p* ≤ 0.05) DNA fragmentation levels in γ-irradiated or carcinogen-treated cells.

**Figure 5:**
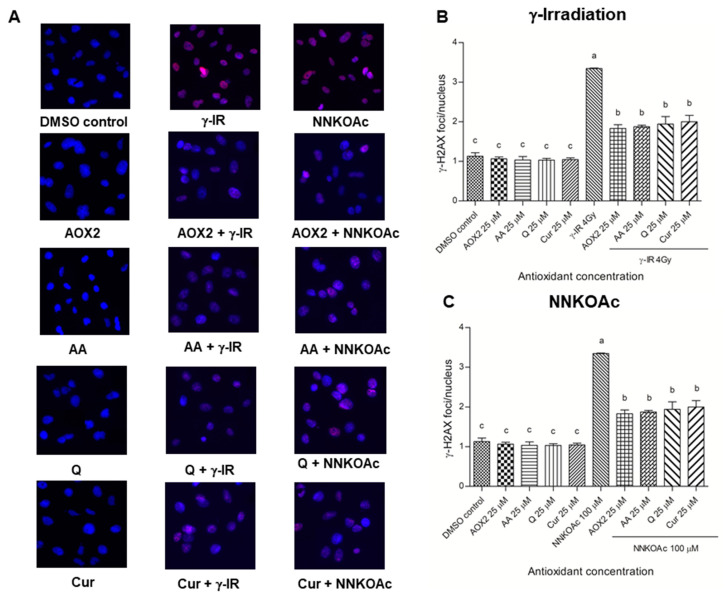
BEAS-2B cells were exposed to γ-irradiation, carcinogen, or in combination with pre-incubation with antioxidants followed by immunofluorescence staining with γ-H2AX antibody and photographed by epifluorescence microscopy at a magnification of 100× (**A**). The nuclei were stained blue, whereas the γ-H2AX foci (S 139) appeared red. Phosphorylated-H2AX level was reduced by pre-exposure to tested antioxidants in γ-irradiation (**B**) or carcinogen (**C**) induced BEAS-2B cells. Quantification of focus/nucleus ratio was calculated for each sample from at least 30 cells. Experimental values are presented as mean ± SD of three independent experiments in triplicate. One-way analysis of variance was performed with Tukey’s pairwise comparison. Means that share the same letter are not significantly different at *p* ≤ 0.05. Abbreviations: AOX2, dietary antioxidant formulation-2; Cur, curcumin; DMSO, dimethyl sulfoxide; γ-IR, γ-irradiation.

Since histone protein serine 139 phosphorylation is a recognized cytological marker of DNA DSBs, the γ-H2AX immunofluorescence assay was used to evaluate DNA damage and the DDR [31]. The nucleus was stained with DAPI and observed under a fluorescence microscope, and the red γ-H2AX foci co-localized with the blue DAPI-stained nucleus were estimated. γ-Irradiated- and NNKOAc-treated cells showed more than 3-times greater DNA damage as compared to the DMSO control cells (Figure 5). When compared to DMSO control cells, pre-treatment with AOX2, AA, Q, or curcumin alone caused no increase in DNA damage; however, their pre-exposure decreased γ-H2AX foci/nuclei induced by γ-irradiation and carcinogen exposure significantly (*p* ≤ 0.05).

**Figure 6:**
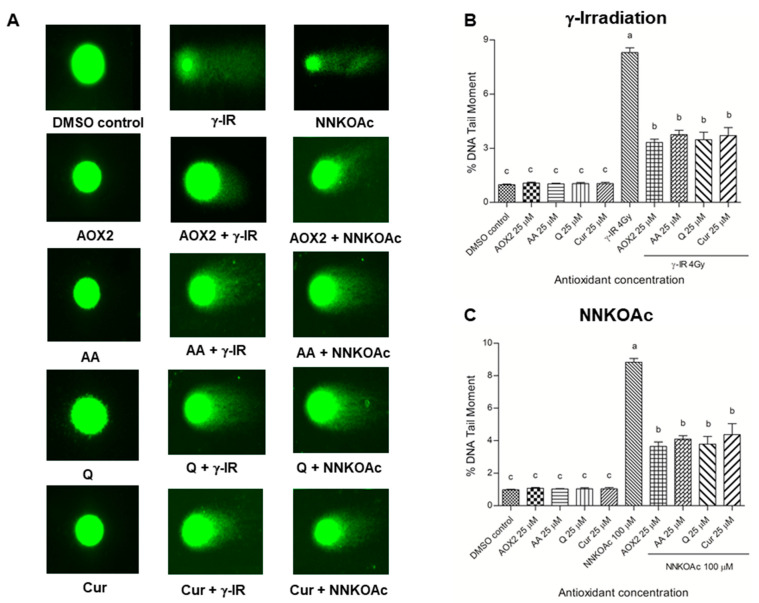
DNA tail damage in BEAS-2B cells that were exposed to γ-irradiation or carcinogen NNKOAc with or without pre-treatment of antioxidants. The cells were stained with CYGREEN^®^ dye and imaged by fluorescence microscopy (**A**). DNA single-strand break was reduced by pre-exposure to tested antioxidants in γ-irradiation (**B**) or carcinogen (**C**) induced BEAS-2B cells. DNA single-strand break was determined as % DNA tail moment measured by comet assay. Quantification was performed for at least 30 comets that were photographed using fluorescence microscopy. Experimental values are mean ± SD of three independent experiments in triplicate. One-way analysis of variance was performed with Tukey’s pairwise comparison. Means that share the same letter are not significantly different at *p* ≤ 0.05. Abbreviations: AOX2, dietary antioxidant formulation-2; AA, ascorbic acid; Q, quercetin; Cur, curcumin; DMSO, dimethyl sulfoxide; γ-IR, γ-irradiation.

A well-known technique for determining DNA damage and repair kinetics in cells is the comet assay, which calculates the “tail moment,” or the tail length × the percentage of fragmented DNA migrating from the nucleus in the tail, during single-cell electrophoresis [32]. The tail-moment in DNA was evaluated after γ-irradiation and carcinogen exposures to the cells with and without dietary antioxidants (Figure 6). Both γ-irradiated and carcinogen-treated cells had about an 8-fold higher tail moment than the controls. AOX2, AA, Q, and curcumin-treated cells had a decreased percentage of fragmented DNA in the tail (*p* ≤ 0.05).

### 3.5. Dietary Antioxidants Affect DDR Cell Signaling

**Figure 7:**
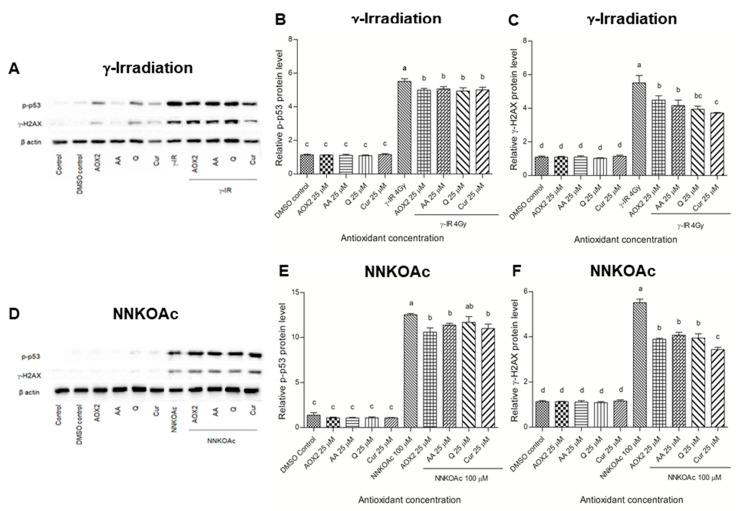
Effect of tested antioxidants on DNA damage repair signaling proteins in BEAS-2B exposed to γ-irradiation (**A**–**C**) or NNKOAc (**D**–**F**). Phosphorylated p53 (p-p53, **B**,**E**) and phosphorylated H2AX (γ-H2AX, **C**,**F**) were assessed by Western blotting. The relative amount of each protein expression level of p-p53 and γ-H2AX was calculated with respect to beta-actin protein. Experimental values are mean ± SD of three independent experiments in triplicate. One-way analysis of variance was performed with Tukey’s pairwise comparison. Means that share the same letter are not significantly different at *p* ≤ 0.05. Abbreviations: AOX2, dietary antioxidant formulation-2; AA, ascorbic acid; Q, quercetin; Cur, curcumin; DMSO, dimethyl sulfoxide; γ-IR, γ-irradiation.

The phosphorylation of tumor suppressor protein p53 and H2AX was also examined and quantified using Western blot analysis (Figure 7). In γ-irradiated and NNKOAc-treated cells, the DDR signaling cascade was boosted, as observed by the levels of p-p53 and γ-H2AX. Pre-treatment with AOX2, AA, Q, or curcumin led to a significant (*p* ≤ 0.05) reduction in phosphorylation of p53 and H2AX in γ-irradiated and NNKOAc-treated cells. Overall, we discovered that pre-treatment with AOX2 and reference antioxidants AA, Q, or curcumin at 25 µM decreased the phosphorylation of these key proteins of DDR cell signaling.

## 4. Discussion

The global environmental radioactive pollution and the radiological impacts on human health are emerging concerns. For example, exposure to ionizing radiation damages DNA, and inefficient repair could lead to mutations, SSB, and DSB DNA damage causing cancer in humans [33,34]. Gamma rays are a type of ionizing radiation that have a high penetrating ability. They can act directly on cellular macromolecules or indirectly on water molecules, causing water radiolysis and subsequently generating free radicals [35]. Secondary consequences of ionizing radiation include ROS generation, which oxidizes proteins and lipids while also causing DNA damage such as apurinic/apyrimidinic sites and SSBs. In addition, mutated extracellular nucleotides, including purinergic signaling, also affect a variety of pathological processes such as inflammation and carcinogenesis [26,36]. All of these alterations, when taken together, result in cell death and mitotic failure [37].

The three primary pathways of NNK metabolism are carbonyl reduction, pyridine nitrogen molecule oxidation, and α-hydroxylation of the methyl or methylene carbons [38]. A substantial part of NNK is transformed into the carcinogenic metabolite 4-(methylnitrosamino)-1-(3-pyridyl)-1-butanol (NNAL), which is then oxidized back to NNK via the carbonyl reduction pathway. Both NNK and NNAL are α-hydroxylated by CYP450 enzymes, resulting in electrophilic intermediates that can interact with DNA to form large pyridyloxobutylation DNA (POB-DNA) adducts [39]. In cell culture, NNKOAc generates α-hydroxymethyl metabolites that spontaneously release 4-3-pyridyl-4-oxobutane-1-diazohydroxide. Diazohydroxides react with DNA generating POB–DNA adducts. In BEAS-2B cells, NNKOAc breaks down into cytosolic reactive electrophilic compounds that cause DNA damage [40]. Therefore, NNKOAc in cultured cells mimics the DNA damage mechanism caused by NNK in vivo [41].

As a result of DNA damage and the inability to repair, it can cause mutations and aberrant cell growth, which transform normal cells into premalignant cells [29]. In this investigation, we established that the AOX2 formulation consisting of 10 well-studied antioxidants can reduce the DNA damage caused by low-dose γ-irradiation and a chemical carcinogen (NNKOAc) in BEAS-2B cells. Previous research has shown that certain vitamins and flavonoids can reduce NNKOAc-induced DNA damage in BEAS-2B cells [21]. Here, we demonstrated that the tested antioxidants do not have cytotoxicity at the efficacious low concentrations, which could be possible to achieve through dietary supplementation. The administration of exogenous ATP in mice and monkeys has shown a radioprotective effect on the lungs [42,43]. However, because ATP is broken down rapidly in living organisms, it can be much more effective in protecting against radiation by regulating concentrations in the lungs using ATP analogs that are not metabolized [26,44].

Clinical data are limited on radioprotective dietary supplements [45]. Cell-based studies and experimental animal models can be employed for the identification and pre-clinical assessment of such dietary supplements. Here, we have optimized an experimental model of low dose γ-irradiation-induced DNA damage using BEAS-2B cells. ROS production is one of the key contributing factors inducing DNA damage in normal healthy cells [46]. In BEAS-2B cells, pre-incubation with AOX2 and the tested single antioxidants significantly reduced the toxic effects of γ-irradiation. Our prior study showed that a vitamin-containing antioxidant formulation (AOX1), apple flavonoids (AF4), quercetin, and quercetin 3-*O*-d-glucoside (Q3G) reduced carcinogen-induced DNA damage [21]. The potential of certain phytochemicals to scavenge ROS is well-known [47]. Ursolic acid prevents cell death by inhibiting free radical generation, lipid peroxidation, oxidative DNA damage, inflammation, and activation of the NF-κB pathway, all of which are caused by radiation [35].

DSBs are among the most complex DNA damages to repair in normal cells. If DSB-induced DNA damage is not repaired, it can lead to genomic instability and, gradually, to tumorigenesis [48]. γ-H2AX phosphorylation is essential for the detection and adoption of cells to DNA damage [49]. Exposure to the radiation and carcinogen used in this study was found to increase γ-H2AX phosphorylation. DNA damage induced by inter stream cross-linking formed by replication forks is observed in γ-irradiated and NNKOAc-treated cells, which is the major cause of γ-H2AX lesions [50]. Furthermore, we used the comet assay to study DNA damage and fragmentation that can detect both SSBs and DSBs [51]. In the present study, we were able to monitor the degree of DNA damage caused by γ-radiation and NNKOAc through quantitative analysis using the properties of comet tail moment. Compared to γ-irradiation/carcinogen treatment, a significant reduction of DNA tail damage was seen when the cells were pretreated with AOX2 and tested antioxidants.

The DDR factor of DNA damage was investigated in this study by Western blotting of two effector proteins, i.e., p53 and γ-H2AX (Figure 7). Activation by phosphorylation of ATM in response to DSB in A549 adenocarcinoma cells followed by phosphorylation of γ-H2AX, Chk2, and p53 has been previously reported [52]. Dietary antioxidants have been shown to possess chemopreventive effects by regulating various signaling pathways, including the p53 pathway. In response to oxidative stress, the activated p53 further activates transcription factors involved in DNA repair, metabolism, aging, apoptosis, autophagy, and angiogenesis [20].

According to a recent study, flavonoids protect Swiss albino mice from ionizing radiation-induced DNA damage in vivo [23]. Celastrol, a triterpene, showed protective effects against γ-radiation-induced DNA damage in Balb/c mice [53]. The study suggested that vitamin C enhances the antiviral activity and also prevents lung fibrosis and injury [54]. Another study found that administration of aerosolized reduced glutathione (GSH) to cystic fibrosis (CF) patients could assist vitamin C status in bronchial epithelia [55]. A recent study demonstrated that quercetin protects BEAS-2B cells from hexavalent chromium (Cr(VI))-induced carcinogenesis by targeting miR-21-PDCD4 signaling [56]. Furthermore, quercetin reduces the lipopolysaccharide-induced release of inflammatory mediators in BEAS-2B cells by increasing cyclic adenosine monophosphate (cAMP) level [57]. As well, curcumin can protect BEAS-2B cells from fine particulate matter (PM_2.5_)-induced oxidative damage and inflammation by activation of NRF2-related pathways to prevent apoptosis [58]. Similarly, the combination of eucalyptol (EUC) and curcumin reduced cigarette smoke extract (CSE)-induced oxidative stress and inflammation to promote cell survival in BEAS-2B cells [59]. Our result showed that the protective effects of AOX2 are similar to those of single antioxidants such as ascorbic acid, quercetin, and curcumin. However, high doses of single antioxidants could act as a prooxidant and may raise the concern of potential toxicity and adverse effects [21].

Phase II metabolism of quercetin is dependent on UDP-glucuronosyltransferases, which are active in UGT1A1, UGT1A8, and UGT1A9, while the rate of conjugation may vary with species and organs [60]. Phase I and II enzymes convert ascorbic acid to oxidized metabolites with higher water solubility and enhanced clearance, which are then used as the electron donor in physiological processes [61]. Folate metabolism is crucial for methylation, reductive processes, and nucleotide production. It also plays a significant part in the carbon pathway [62]. The metabolism of vitamin B12 is a complicated process; however, deoxyadenosylcobalamin and methylcobalamin are two major metabolites [63]. Two processes that make up lipoic acid metabolism are interdependent and unable to fully compensate for each other, and the first lipoylation of the H-protein in the glycine cleavage system (Gcv3) is necessary [64]. The response to dietary and medicinal interventions varies significantly across individuals depending on how α-tocopherol is metabolized. The various metabolites of α-tocopherol have been discovered in human blood, which gives rise to the potential of exploring the variability, known as “vitamin E metabolome” [65]. A limited number of investigations have been done on CoQ10 metabolism. The metabolites were primarily discovered in the urine and were also noted in the feces, where significant amounts of non-metabolized CoQ10 were discovered [66]. Zeaxanthin, which is mostly found in the eyes, skin, and brain, has not yet been studied in terms of its metabolism [67]. In primary rat hepatocytes, astaxanthin can be metabolized into 3-hydroxy-4-oxo-ionone and 3-hydroxy-4-oxo-7,8-dihydro-ionone. Enzymes that catalyze the formation of the metabolites have not yet been fully understood, nor have their potential biological activities. Astaxanthin raises cytochrome P450 (CYP) enzyme levels in hepatocytes [68]. Selenium (Se) is a vital micronutrient with many beneficial effects including antioxidant properties. In general, selenium is well tolerated by organisms through bioaccumulating and converting to selenoproteins. The glutathione metabolism pathway plays a significant role in selenium metabolism [69].

The ultimate goal of this research was to develop an enhanced natural dietary antioxidant formula or dietary supplement which can reduce the risk of cancer by exposure to diagnostic radiation and chemical carcinogens. As such, dietary antioxidant formulations could help in reducing the growing burden in the healthcare system from radiation and carcinogen-induced cancers. Our findings indicate that dietary antioxidants and phytochemical preparations such as AOX2 can protect against DNA damage in normal bronchial BEAS-2B cells from exposures to radiation and smoking-related carcinogens, which warrants further exploration using pre-clinical animal models to further understand the oncoprotective properties of AOX2.

## 5. Conclusions

This study shows that pre-exposure of BEAS-2B cells to the AOX2 dietary antioxidant formulation protects the cells from low dose γ-radiation or carcinogen NNKOAc. However, the present findings need to be validated using pre-clinical animal and human clinical studies before recommending dietary antioxidants as therapeutic supplements to reduce the risk of cancer due to exposure to carcinogenic environmental factors, genotoxic diagnostic radiation, or chemotherapy.

## Data Availability

Data is contained within the article.

## Abbreviations

AA: ascorbic acid; AOX2, dietary antioxidant formulation-2; BEAS-2B, normal human bronchial epithelial cells; Cur, curcumin; DDR, DNA damage response; DMSO, dimethylsulfoxide; DSB, DNA double-strand breaks; FA, folic acid; IR, irradiation; NNK, 4-[(methyl)nitrosamino]-1-(3-pyridyl)-1-butanone; NNKOAc, 4-[(acetoxymethyl)nitrosamino]-1-(3-pyridyl)-1-butanone; Q, quercetin; ROS, reactive oxygen species.
